# Homologous production, one-step purification, and proof of Na^+^ transport by the Rnf complex from *Acetobacterium woodii*, a model for acetogenic conversion of C1 substrates to biofuels

**DOI:** 10.1186/s13068-020-01851-4

**Published:** 2020-12-21

**Authors:** Anja Wiechmann, Dragan Trifunović, Sophie Klein, Volker Müller

**Affiliations:** Molecular Microbiology and Bioenergetics, Institute of Molecular Biosciences, Goethe University Frankfurt, Max-von-Laue Str. 9, 60438 Frankfurt am Main, Germany

**Keywords:** Anaerobic bacteria, Respiration, Energy conservation, Sodium transport

## Abstract

**Background:**

Capture and storage of the energy carrier hydrogen as well as of the greenhouse gas carbon dioxide are two major problems that mankind faces currently. Chemical catalysts have been developed, but only recently a group of anaerobic bacteria that convert hydrogen and carbon dioxide to acetate, formate, or biofuels such as ethanol has come into focus, the acetogenic bacteria. These biocatalysts produce the liquid organic hydrogen carrier formic acid from H_2_ + CO_2_ or even carbon monoxide with highest rates ever reported. The autotrophic, hydrogen-oxidizing, and CO_2_-reducing acetogens have in common a specialized metabolism to catalyze CO_2_ reduction, the Wood–Ljungdahl pathway (WLP). The WLP does not yield net ATP, but is hooked up to a membrane-bound respiratory chain that enables ATP synthesis coupled to CO_2_ fixation. The nature of the respiratory enzyme has been an enigma since the discovery of these bacteria and has been unraveled in this study.

**Results:**

We have produced a His-tagged variant of the ferredoxin:NAD oxidoreductase (Rnf complex) from the model acetogen *Acetobacterium woodii*, solubilized the enzyme from the cytoplasmic membrane, and purified it by Ni^2+^–NTA affinity chromatography. The enzyme was incorporated into artificial liposomes and catalyzed Na^+^ transport coupled to ferredoxin-dependent NAD reduction. Our results using the purified enzyme do not only verify that the Rnf complex from *A. woodii* is Na^+^-dependent, they also demonstrate for the first time that this membrane-embedded molecular engine creates a Na^+^ gradient across the membrane of *A. woodii* which can be used for ATP synthesis.

**Discussion:**

We present a protocol for homologous production and purification for an Rnf complex. The enzyme catalyzed electron-transfer driven Na^+^ export and, thus, our studies provided the long-awaited biochemical proof that the Rnf complex is a respiratory enzyme.

## Background

Currently, mankind faces the problem to sustain the energy demand for society and to combat global warming. Both problems are ultimately linked, since conventional energy production by, for example, burning fossil fuels leads to the production of carbon dioxide that increases global warming [1, 2]. Future energy demands require alternatives for the current energy technologies that are based mainly on fossil fuels [3]. Hydrogen is a promising and widely considered alternative energy provider and a hydrogen-based economy is a prime candidate for a sustainable future without the need of fossil fuels. A striking problem of using hydrogen as energy source is the storage and transport due to the physical properties of this highly reactive gas [4, 5]. If hydrogen storage can be coupled to CO_2_ reduction, two birds are killed with one stone. Indeed, chemical catalysts for hydrogen storage are mostly based on binding hydrogen-to-carbon dioxide [6]. The same is done by autotrophic, non-phototropic, CO_2_-reducing, hydrogen-oxidizing anaerobic bacteria, and archaea [7]. One group, the acetogenic bacteria, forms acetate from two molecules of CO_2_ and four molecules of H_2_ [7–9]. In addition to acetate, some acetogens can produce ethanol or other alcohols [10–12]. Although this route is suitable for capturing carbon dioxide or carbon monoxide from waste gas streams, it is not suitable for storage of hydrogen, since the backwards reaction starting from acetate is thermodynamically difficult [13]. However, hydrogen can be stored by these bacteria in the form of formic acid in a reaction that is close to thermodynamic equilibrium and thus suitable for hydrogen storage and production via formic acid as intermediate [14, 15].

**Fig. 1:**
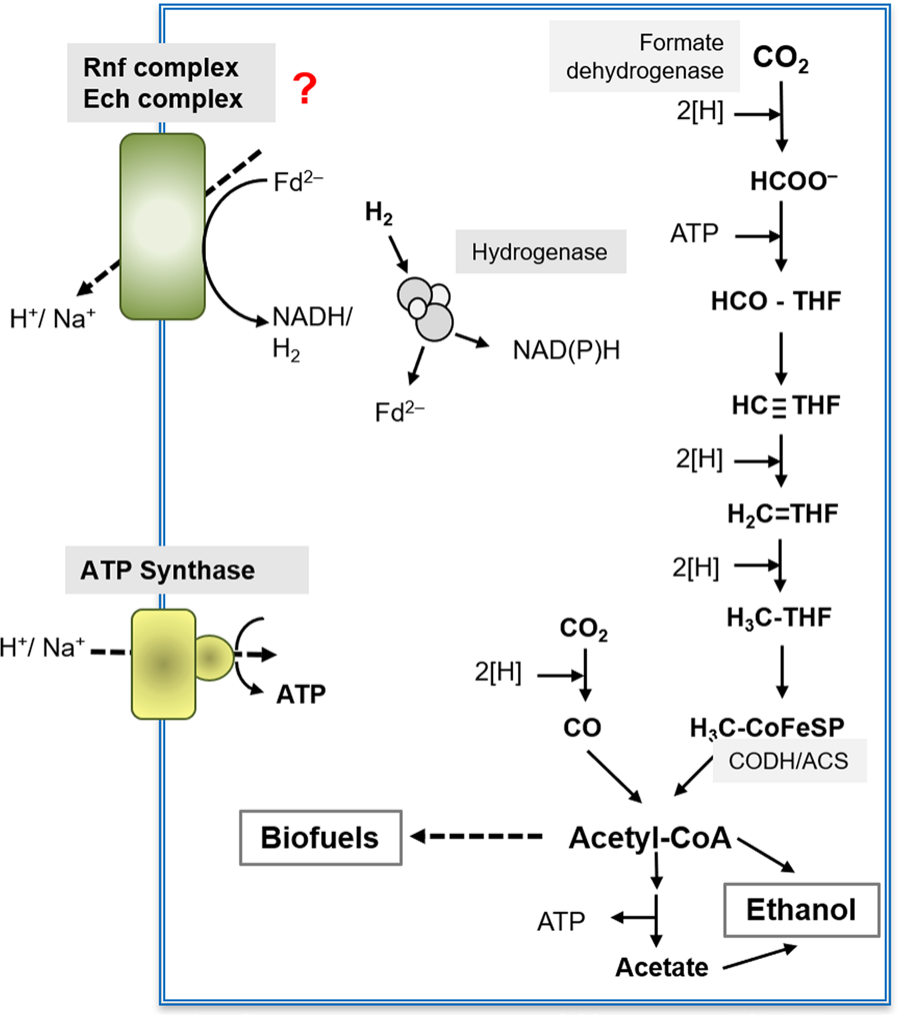
Schematic overview of potential biofuel formation during acetogenesis from H_2_ + CO_2_ in acetogenic bacteria using either the Rnf or Ech complex. [H], reducing equivalent; Fd^2−^, reduced ferredoxin; CODH/ACS, carbon monoxide reductase/acetyl-CoA synthase; THF, tetrahydrofolate; CoFeSP, corrinoid iron–sulfur protein. Ion transport and redox reactions are not stoichiometric

Acetogenic bacteria are ubiquitous in nature and grow chemolithoautotrophically by converting H_2_ + CO_2_ to acetate [7, 9, 16, 17]. CO_2_ is reduced by the Wood–Ljungdahl pathway, the only pathway of CO_2_ fixation that has no demand for additional ATP and, thus, is often considered as ancient, if not being the first biochemical pathway on Earth [18]. One molecule of ATP is produced by the pathway, but one is also consumed (Fig. 1). How net ATP is synthesized in these bacteria has been an enigma since their discovery. Experiments with whole cells and subcellular fractions are all in line with the hypothesis that acetogens employ either one of the two potential respiratory complexes Ech (energy-converting hydrogenase), a ferredoxin:H^+^ oxidoreductase and Rnf (*Rhodobacter* nitrogen fixation), a ferredoxin:NAD oxidoreductase for electron transport phosphorylation [16, 17, 19–23], but the final biochemical proof for respiration (ion translocation) requires the purification of the enzyme followed by its reconstitution into artificial liposomes and reconstitution of ion transport into the artificial lipid droplets. These experiments were unsuccessful so far, since neither the Ech nor Rnf complex has been able to be purified from an acetogenic bacterium nor the heterologous production and purification of the Rnf complex of *A. woodii* in *E. coli* resulted in a functional enzyme [24]. Clearly, a heterologous expression in *Escherichia coli* is hampered by the fact that the enzyme not only requires iron-sulfur centers but also covalently-linked flavins and a homologous expression system would be highly appreciated. In this study, we took advantage of the recently generated *rnf*-deletion mutant of the model acetogen *Acetobacterium woodii* [24]. We describe a procedure for the expression of the *rnf* genes *in trans* which complements the mutation. Furthermore, using a tagged version the enzyme was purified to apparent homogeneity in one step. We will provide evidence that the purified enzyme is an electrogenic and primary sodium ion pump providing the final biochemical proof that the Rnf complex of *A. woodii* is a Na^+^-translocating respiratory enzyme that uses the energy of electron transfer from ferredoxin (E_O_’ = − 450 to − 500 mV) to NAD (E_O_’ = − 320 mV) [17], to expel sodium ions from the cell and thus energize the membrane for ATP synthesis.

## Results and discussion

### Expression of a functional Rnf complex in  *A. woodii*

**Fig. 2:**
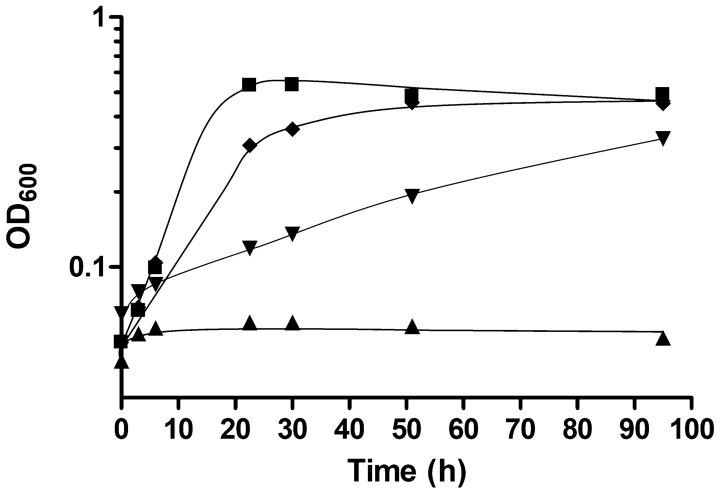
Growth restoration of the *rnf* mutant on H_2_ + CO_2_ after complementation with the plasmid pMTL_8312_Ptet_SrnfH. The *A. woodii* wild type (■), the wild type containing the vector pMTL8312_Ptet without the *rnf* operon (♦), the *rnf* mutant (▲), and the complemented strain Δ*rnf* pMTL_8312_Ptet_SrnfH (▼) were grown in complex medium under a H_2 _+ CO_2_ [80:20 v/v] atmosphere with a pressure of 1.0 × 10^5^ Pa. Plasmid-containing strains were induced with 400 ng/ml atet at an OD_600_ of 0.8–1.1. The OD_600_ was followed over 95 h. *N* = 2 independent experiments

To express the genes *rnfCDGEAB,* they were first amplified from chromosomal DNA of the wild type of *A. woodii* and cloned into the vector pMTL_83121 [25] under the control of a TetR repressor/promoter system [26]. The vector is a modular shuttle vector containing a chloramphenicol resistance cassette, a Gram^+^ replicon (originated from plasmid pCB102 from *Clostridium butyricum*) and a Gram^−^ replicon (p15A) [25] in which the TetR repressor/promoter system was cloned according to Beck *et al.*, 2020 [26]. Sequences encoding one strep tag and one His-tag were introduced at the 5′ end of *rnfC* and the 3′ end *rnfB*, respectively, resulting in plasmid pMTL_8312_Ptet_SrnfH. The *A. woodii* wild type was transformed with the plasmid and grown on fructose as carbon and energy source in the presence of chloramphenicol to select for recombinants. Plasmids were isolated from the recombinants and proven to be unaltered in *A. woodii* by restriction mapping. Next, we analyzed whether the plasmid would complement the phenotype of the Δ*rnf* mutant. Therefore, wild type, wild type containing pMTL_8312_Ptet without the *rnf* genes, Δ*rnf* mutant, and complemented mutant were grown under different conditions. The wild type, the wild type plus pMTL_8312_Ptet grew on fructose or H_2_ + CO_2_, but the Δ*rnf* mutant did not grow on H_2_ + CO_2_, as seen before [20]. The mutant was then complemented *in trans* with the plasmid pMTL_8312_Ptet_SrnfH and the complemented mutant grew on H_2_ + CO_2_, but with a lower final yield (65% and 75%) compared to the wild type and wild type plus pMTL_8312_Ptet, respectively (Fig. 2). The doubling time of approx. 32 h was also much slower than for the wild type (6 h) and wild type plus pMTL_8312_Ptet (9.5 h). This experiment demonstrates that a functional Rnf complex can be produced in the complemented Δ*rnf* mutant.

### One-step purification of homologously produced functional Rnf complex in  *A. woodii*

To produce the Rnf complex in *A. woodii*, cells of *A. woodii* Δ*rnf* (pMTL_8312_Ptet_SrnfH) were grown on fructose as carbon and energy source to an OD_600_ of 0.3, and then, gene expression was induced by addition of anhydrotetracycline (aTet) to a final concentration of 400 nM [26]. After incubation for ~ 24 h post-induction, cells were harvested, and cell extract was prepared under strictly anoxic conditions. The cytoplasmic membrane was separated from the cell extract by ultracentrifugation. Western blot analyses with antibodies available against RnfB, RnfG, or RnfC demonstrated that the proteins were present in the membrane fraction (data not shown). Membranes were resuspended in buffer A1 (50 mM Tris–HCl, pH 8.0, 20 mM MgSO_4_, 2 mM DTE, and 4 µM resazurin), solubilized by addition of 1 mg DDM (*n*-dodecyl-*β*-D-maltopyranoside) per mg of protein for 120 min at 4 °C, and the supernatant was applied to a 4 ml Ni^2+^-NTA or Streptactin column. After using the Strep-Tactin column, no proteins could be detected in the elution fractions. Proteins were only detected in the flow-through, showing that the N-terminal Strep-tag attached to RnfC did not bind to the Strep-Tactin material. Attempts to purify the Rnf complex using a Ni^2+^-affinity matrix were more successful. However, with a His-tag attached to RnfB (C-terminal) proteins could be purified, but in insufficient amounts which made further studies with the protein complex impossible. Therefore, we inserted a second His-tag (C-terminal on RnfG). When using the strain Δ*rnf* (pMTL_8312_Ptet_rnf) which contained His-tags on two positions (Additional file 1: Sequence S1), the C-terminus of RnfB and RnfG, sufficient amounts of the Rnf complex could be purified *via* a Ni^2+^ column.

The membrane fraction had a ferredoxin:NAD oxidoreductase (FNO) activity of 73 mU/mg which increased by a factor of 4.5 to 322 mU/mg in the solubilizate. Fractions were eluted from the Ni^2+^ column in 4 ml steps in elution buffer (50 mM Tris–HCl, 150 mM imidazole, pH 8.0, 20 mM MgSO_4_, 0.02% [w/v] DDM, 5 μM FMN, 2 mM DTE, and 4 µM resazurin). FNO activity eluted over several fractions with specific activities of 5.8–7.3 U/mg. Highest activity was detected in elution fractions E2 and E3, with 6.6 and 7.3 U/mg, respectively. These two fractions were pooled. The total amount of protein was 3.7 mg from a 4 l culture and the total FNO activity was 25.7 U.

**Fig. 3:**
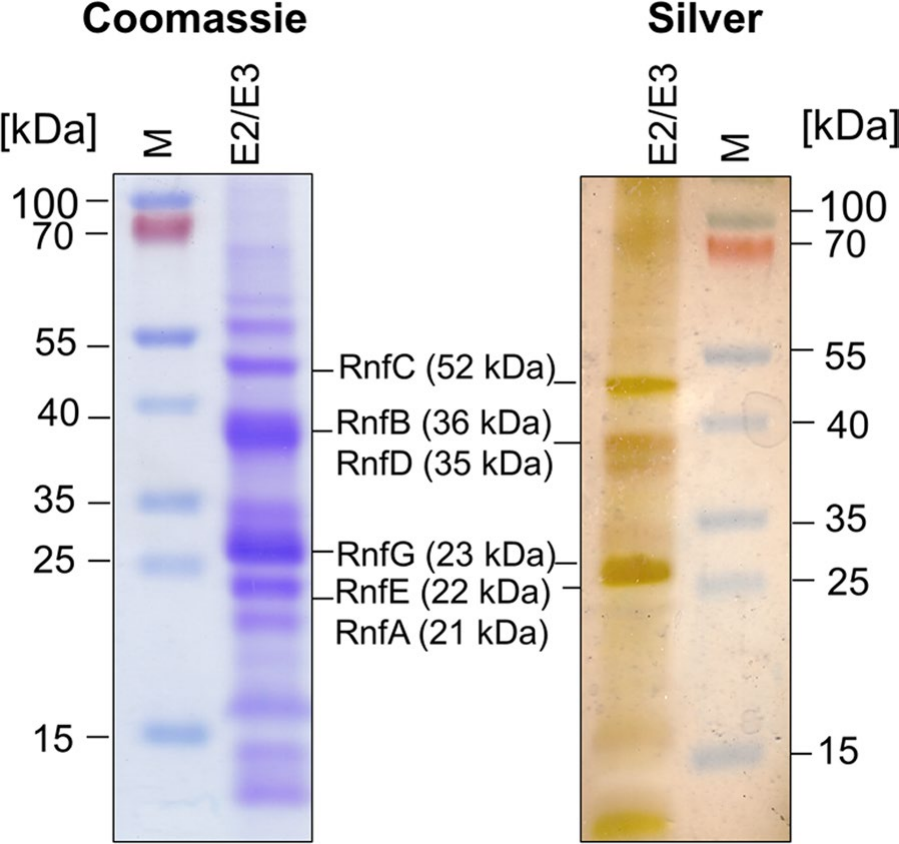
Purification of the overproduced Rnf complex from *A. woodii* in *A. woodii* Δ*rnf*. *A. woodii* Δ*rnf* + pMTL8312_Ptet_rnf was grown at 30 °C in complex medium. After reaching an OD_600_ of ~ 0.2–0.3, Rnf production was induced with 400 nM aTet. Cells were harvested, crude extract was prepared, and membranes were isolated by ultracentrifugation. After solubilization with  DDM, proteins were purified using a Ni^2+^-affinity column. Elution fractions E2 and E3 were pooled and proteins (20 µg) were separated on an SDS-PAGE and either stained with Coomassie Brilliant Blue or silver. M, molecular mass standard

**Fig. 4:**
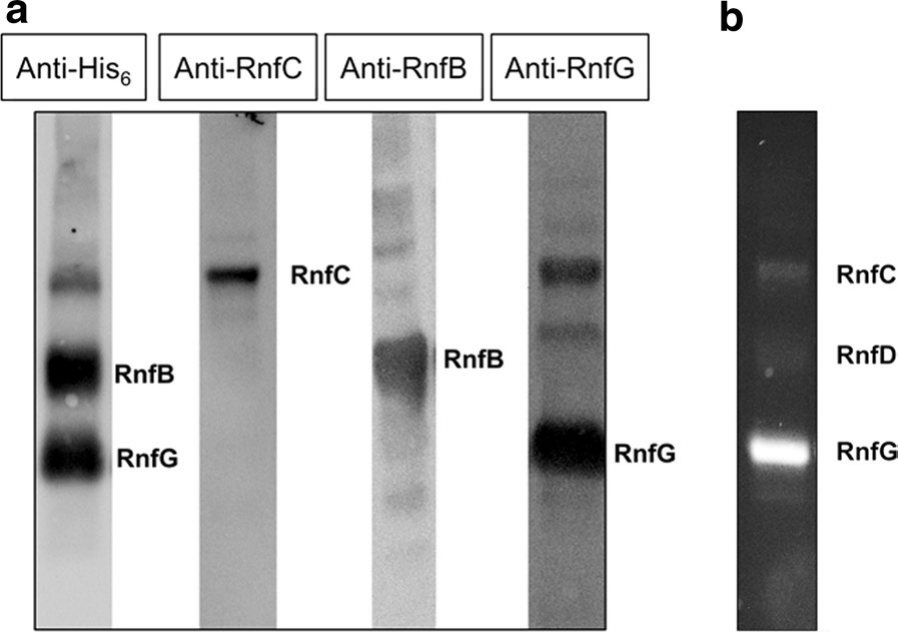
Western Blot analysis of Rnf subunits and detection of FMN in Rnf subunits of the purified Rnf complex from *A. woodii.* Purified proteins (20 µg) were separated on an SDS-PAGE and were either transferred onto nitrocellulose membranes and blotted against His_6_, RnfC, RnfB, and RnfG antibodies (**a**) or were placed under UV light (280 nm) for visualization of flavin-containing subunits (**b**)

The proteins present in the preparation were analyzed by SDS-PAGE. As can be seen in Fig. 3, proteins with molecular masses of ~ 50 kDa, 38 kDa, 30 kDa, and 20 kDa were seen. The 50 kDa protein corresponds in size to RnfC (52 kDa), the broad band at around 38 kDa could harbor RnfD (35 kDa) and RnfB (36.6 kDa), the 30 kDa protein corresponds to RnfG, and the band only visible after coomassie staining at 20 kDa could harbor RnfA (21.4 kDa) and RnfE (21.6 kDa). The identity of RnfC, RnfB, and RnfG were confirmed by western blotting using specific antibodies (Fig. 4a) [27, 28]. Furthermore, His_6_-RnfB and His_6_-RnfG reacted with an antibody against the His-tag (Fig. 4a). The membrane-integral subunits RnfD and RnfA for which antibodies are not available were identified by peptide mass fingerprinting (PMF), (Additional file 2: Figure S1). RnfD and RnfG were shown to have a covalently bound FMN, and accordingly, RnfD and RnfG showed fluorescence typical for flavins under UV light (Fig. 4b). As did RnfC, which was first proposed to contain a flavin, since potential FMN-binding sites were found in the sequence of RnfC [29]. However, flavins have not been detected in RnfC experimentally [27] and a recent study could not confirm the presence of FMN in RnfC of *A. woodii*, heterologously produced in *E. coli* [25]*.* In contrast, our studies clearly show fluorescence of a potential flavin in RnfC, suggesting that a flavin might be covalently bound to this subunit. RnfE was not detected by MALDI-TOF analysis, but this could be due to its hydrophobic nature. Anyway, we suspect it to be present in the preparation and, if not, it is dispensable for electron transfer and Na^+^ transport, but this seems to be unlikely.

**Fig. 5:**
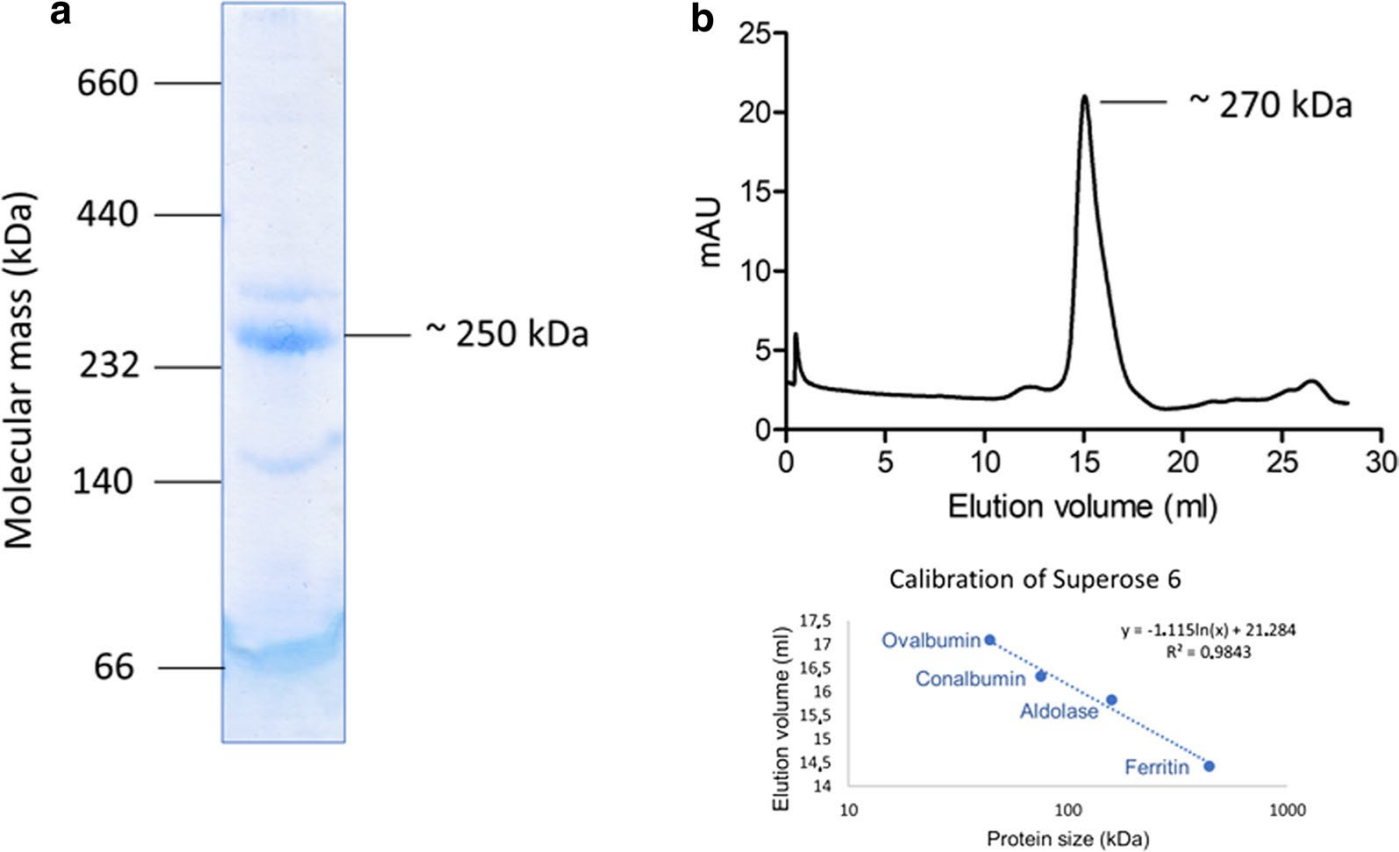
Size determination of the purified Rnf complex of *A. woodii*. After purification via a Ni^2+^ column, 10 µg of the purified Rnf complex were loaded on a native polyacrylamide gel and stained with Coomassie Brilliant Blue (**a**) and 340 µg of the complex were loaded onto a Superose 6 column (**b**), which was eluted in buffer-containing 50 mM Tris–HCl, pH 8.0, 20 mM MgSO_4_, 5 µM FMN, 0.02% DDM, 2 mM DTE, and 4 µM resazurin, respectively

The quaternary structure of the Rnf complex was analyzed by native gel electrophoreses and gel filtration which revealed masses of ~ 250 kDa and ~ 270 kDa, respectively (Fig. 5a and b). Assuming one copy of each subunit, the complex would have a calculated mass of 198 kDa. The divergence of the apparent mass by 25–35% may arise from the detergent micelles, and a monomeric state is more likely than a dimeric state (396 kDa).

In addition to the Rnf subunits other proteins such as subunits of the Na^+^-dependent F_1_F_O_ ATP synthase, V_1_V_O_ ATPase, alcohol dehydrogenase AdhE, and lysozyme were found by MALDI-TOF in the preparation. The Na^+^-F_1_F_O_ ATP synthase activity was very low (88 mU/mg), and only AtpD and AtpF which were found by MALDI-TOF were also detected by Western blotting when using specific antibodies against AtpD, AtpF, AtpB, and AtpE, ruling out a functional Na^+^-F_1_F_O_ ATP synthase in the preparation. Anyway, a physical interaction of Na^+^-Rnf and Na^+^-F_1_F_o_ ATP synthase was observed before [30] and may point to a respiratory supercomplex.

### The Rnf complex is a Na^+^-dependent, primary, and electrogenic Na^+^ pump

**Fig. 6:**
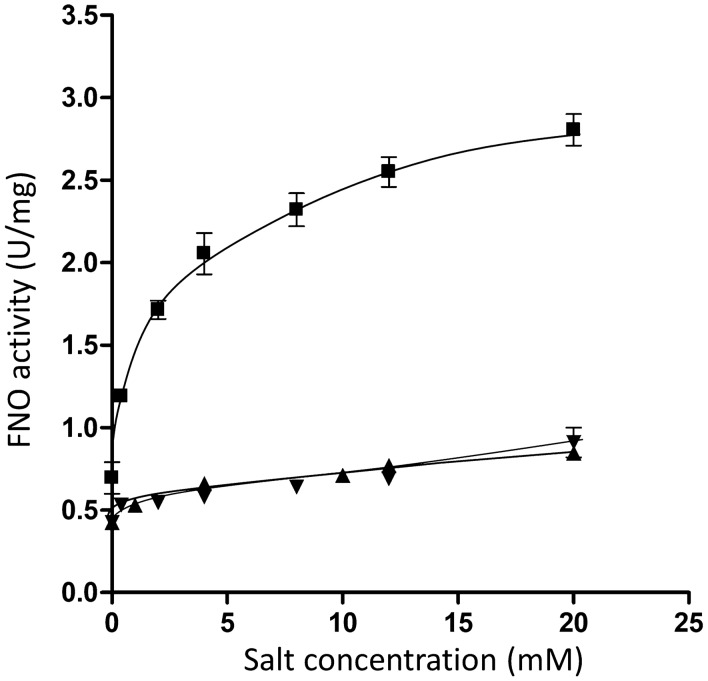
Na^+^ dependence of ferredoxin:NAD oxidoreductase (FNO) activity as catalyzed by the purified Rnf complex. Dependence of FNO activity on different concentrations of NaCl (■), KCl (▼), or LiCl (▲) was measured in anoxic cuvettes containing Na^+^-free buffer (20 mM Tris–HCl, pH 7.7, 2 mM DTE, 4 µM resazurin; contaminating Na^+^ concentration: 0.125 µM Na^+^) under a CO atmosphere with a pressure of 0.5 × 10^5^ Pa. 10 µl ferredoxin (3 mM), 5 µl carbon monoxide dehydrogenase (CODH; 30 mg/ml), and 14 µg of the Rnf complex were added. The reaction was started by addition of 30 µl NAD^+^ (100 mM) and reduction of NAD^+^ at 340 nm was measured. Data points represent two replicates

**Fig. 7.**
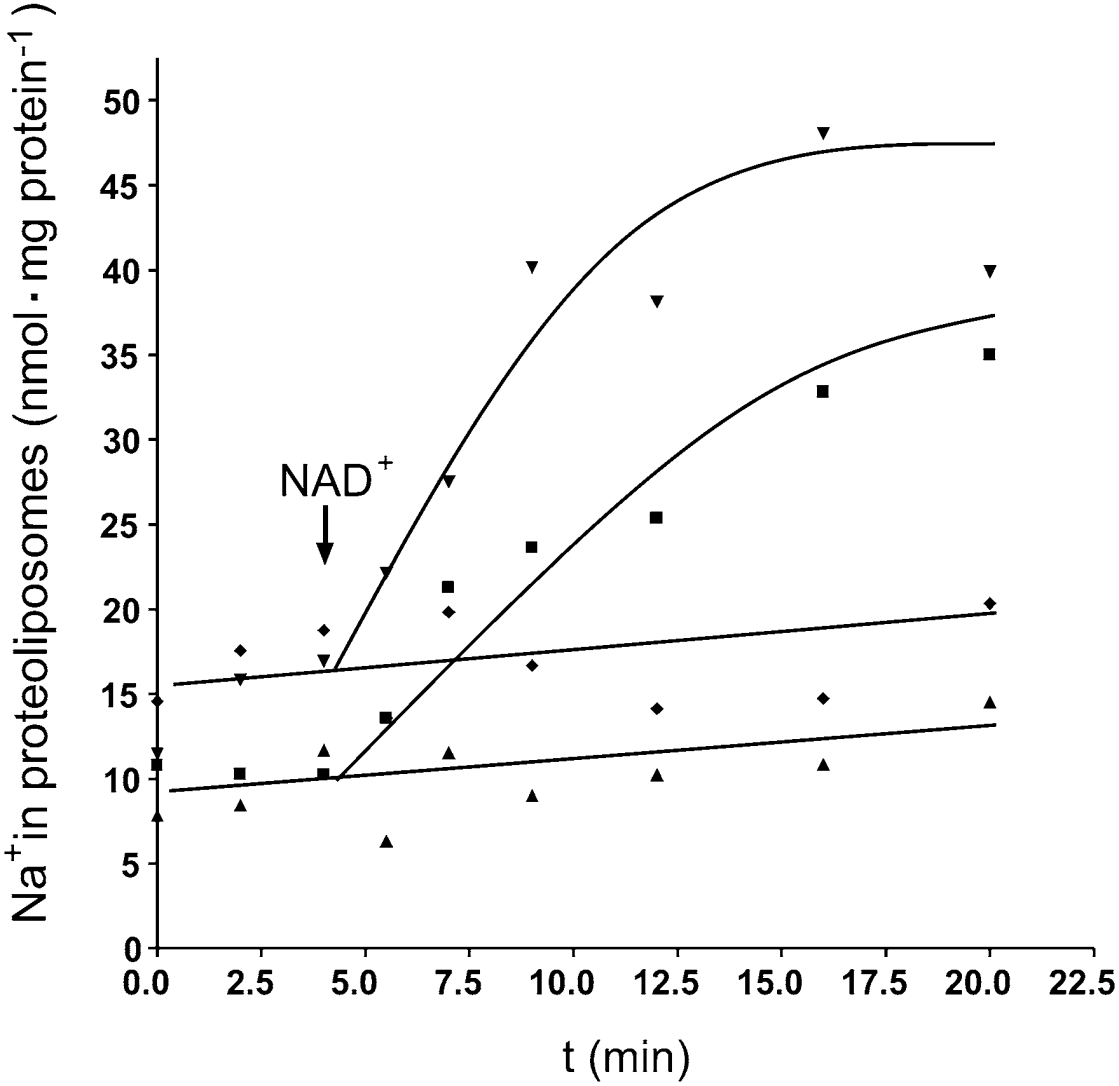
^22^Na^+^ transport by the Rnf complex reconstituted into proteoliposomes. 220 µl proteoliposomes (protein concentration 2.84 mg/ml) in buffer (100 mM Tris–HCl, 100 mM malic acid, pH 7.4, 5 mM MgCl_2_, 2 mM NaCl, 2 mM DTE, and 4 μM resazurin) catalyzed ^22^Na^+^ transport upon addition of 10 μl ferredoxin (3 mM), 10 μg CODH (3 mg/ml), and 30 μl NAD^+^ (100 mM) under anoxic conditions with CO in the headspace (■). One assay contained 40 µM of the protonophore TCS (▼). Another 40 µM of the Na^+^-ionophor ETH2120 (◆). One assay did not contain NAD^+^ (▲). Data points show one representative out of two biological replicates

The purified Rnf complex catalyzed the physiological activity of oxidation of reduced ferredoxin with reduction of NAD, demonstrating that the electron transport chain is intact. To prove intactness of the membrane domain and its coupling to the electron transport chain, we analyzed the sodium ion dependence of electron transport. As shown in Fig. 6, ferredoxin:NAD oxidoreductase activity was marginal in the absence of Na^+^ (contaminating concentration 0.125 mM), but increased with increasing NaCl concentrations. Half maximal activity was obtained at 1 mM NaCl. KCl and LiCl did not stimulate activity, demonstrating a strict Na^+^ dependence of electron transport. To finally prove Na^+^ transport by the enzyme, it was reconstituted under strictly anoxic conditions into liposomes made from phosphatidyl choline from soybeans. The Rnf-containing liposomes were incubated in buffer-containing ferredoxin, the ferredoxin-reducing system, and ^22^Na^+^ [30]. Upon addition of the electron acceptor NAD^+^, electron transport from reduced ferredoxin started, and concomitantly, Na^+^ was transported into the lumen of the vesicles with a rate of 1.58 ± 0.3 nmol/mg min (Fig. 7). When NAD^+^ was omitted, there was no ^22^Na^+^ transport. ^22^Na^+^ transport leads to an accumulation of positive charges inside the proteoliposomes, and thus, charge compensation should stimulate ^22^Na^+^ transport; this was indeed observed; the rate of ^22^Na^+^ transport increased slightly in the presence of the protonophore 3,3′,4′,5'-tetrachlorosalicylanilide (TCS). Accumulation of ^22^Na^+^ was completely prevented by the sodium ionophore ETH2120 (Fig. 7). In summary, these experiments revealed that the Rnf complex catalyzes a primary and electrogenic Na^+^ transport.

## Conclusion

The homologous expression system for a strict anaerobe described here allows for quick and mild purification of a functional heterooligomeric membrane protein harboring several unusual cofactors that could not be achieved using a heterologous expression system in *E. coli*. Using this methodology, we could provide the final and long-awaited proof that the Rnf complex of the acetogenic model bacterium *A. woodii* is a Na^+^-translocating respiratory enzyme. This finding extends the spectrum of known respiratory enzymes to the far electronegative side (E_0_′ = − 450 to − 350 mV) and unraveled the respiratory enzyme in one group of acetogens, the Rnf-containing acetogens. Acetogens grow at the thermodynamic edge of life [17] and production of industrially interesting biofuels is thermodynamically restricted, i.e., due to a low ATP yield [31]. With the discovery made here, we may now overcome energetic barriers in acetogenic C1 conversions by optimizing Rnf activity or by implementing additional reactions leading to the reduction of ferredoxin, the fuel for chemiosmotic ATP synthesis, required to drive biosynthetic pathways.

## Methods

### Bacterial strains and growth conditions

For construction of plasmids, *Escherichia coli* HB101 (Promega, Madison, WI, USA) was used. *A. woodii* DSM1030 lacking the *rnf* operon [24] was grown in complex medium at 30 °C under anaerobic conditions as previously described [32–34], and only that 50 µg/ml uracil were added. 20 mM fructose was used as carbon source. *E. coli* was cultivated in lysogeny broth (LB) medium [35]. Chloramphenicol for use in *E. coli* (30 µg/ml) or its derivative thiamphenicol for use in *A. woodii* (35 µg/ml) was added appropriately.

### Construction of pMTL_8312_Ptet_rnf and transformation of *A. woodii*

All primers are listed in Table 1. The expression vector pMTL_8312_Ptet_rnf was constructed by firstly amplifying nucleotides coding for the Tet-repressor following its target promoter region using primers G_Ptet_for and G-Ptet_rev [26, 36], following a PCR of the vector pMTL83121 (containing a chloramphenicol resistance cassette, the Gram^−^ p15A origin of replication, and the Gram^+^ pCB102 origin of replication) with primers G_pMTL8312_for and G_pMTL_8312_rev. The amplified products were ligated via Gibson assembly using the HiFi DNA Assembly Kit (NEB, Frankfurt am Main, Germany), allowing the *tetR* and *tet* promoter region to insert into the *Not*I and *Nde*I sites of the MCS of pMTL83121, resulting in pMTL8312_Ptet. After construction of pMTL8312_Ptet, the *rnf *operon of *A. woodi* was amplified with primers G_SrnfH_for and G_SrnfH_rev using the already existing plasmid pT7-7_rnf [28], which contained the sequences for a strep-tag at the N-terminus of RnfC and for a His-tag at the C-terminus of RnfB. These were ligated into pMTL8312_Ptet which was amplified with primers G_pMTL_tet_for and G_pMTL_tet_rev via the HiFi DNA Assembly Kit (NEB, Frankfurt am Main, Germany), resulting in pMTL8312_Ptet_SrnfH. For addition of the sequence coding for His-tag at the C-terminus of RnfG primers RnfG_His_for and RnfG_His_rev, the Q5® Site-Directed Mutagenesis Kit was used (NEB, Frankfurt am Main, Germany). The resulting plasmid pMTL_Ptet_rnf was checked by sequencing analysis (Microsynth Seqlab, Göttingen, Germany) before it was transformed into the *A. woodii* Δ*rnf* mutant [24]. Competent cells of the mutant were generated as previously described [37], only that the *A. woodii* Δ*rnf* mutant was grown in complex medium as described in the above section. To verify a correct transformation, plasmids were isolated from the *A. woodii* Δ*rnf* mutant using the Zippy Plasmid Miniprep Kit (Zymo Research Europe GmbH, Freiburg, Germany). Plasmids were retransformed into *E. coli* HB101, plasmids were again isolated and checked by sequencing analysis.

**Table 1:** Primers used in this study

Primer name	Sequence 5′→3′
G_Ptet_for	ACAGCTATGACCGCGGCCGCttaagacccactttcacatttaag
G_Ptet_rev	TTCGTAATCATGGTCATatgagttaacctcctgtcgac
G_pMTL8312_for	catatgaccatgattacgaattc
G_pMTL8312_rev	gcggccgcggtcatagct
G_SrnfH_for	TCGTTAGTCGACAGGAGGTTAACTCATatgtggagccacccgcagttc
G_SrnfH_rev	GCTAGCGCCATTCGCCATTCAGGCTGCGCActaatggtgatggtgatggtgtttgg
G_pMTL_tet_for	tgcgcagcctgaatggcg
G_pMTL_tet_rev	catatgagttaacctcctg
RnfG_His_for	**catcaccatcaccat**taggagggaaaagtaaagatg
RnfG_His_rev	**gtg**ggcgcctttcgacaaattctggtaaac

Underlined letters specify restriction sites, capital letters highlight the homologous extensions for Gibson assembly, and bold letters are extension sited for addition of an His-tag via site-directed mutagenesis

### Production and purification of the Rnf complex

*A. woodii* Δ*rnf* containing pMTL_Ptet_rnf was grown in 4 l complex medium containing 30 µg/ml thiamphenicol until reaching an OD_600nm_ of 0.3. Production of the Rnf complex by *A. woodii* Δ*rnf* pMTL_Ptet_rnf was induced by addition of 400 ng of anhydrotetracycline (aTet) [26] and cells were harvested 24 and (Coy Laboratory Products, Gras Lake Charter Township, MI, USA) 25 mM Tris–HCl, pH 7.8, 420 mM sucrose, 2 mM DTE, and 4 µM resazurin) and resuspended in 30 ml lysis buffer before cells were treated with 2.8 mg/ml lysozyme for 1 h. Protoplasts were then separated from lysozyme via centrifugation (12,000 ×*g*, 10 min, 4 °C) and were resuspended in 20 ml buffer A1 (50 mM Tris–HCl, pH 8.0, 20 mM MgSO_4_, 2 mM DTE, and 4 µM resazurin). 1 mg/ml DNaseI and 1 mM PMSF (phenylmethylsulfonyl fluoride) were added and protoplasts were passed twice through a French pressure cell at a pressure of 110 MPa. The cell crude extract was separated from cell debris by centrifugation at 25,000×*g* for 20 min and membranes were further separated from the cytosol by ultracentrifugation (130,000 ×*g*, 1 h, 4 °C). Membranes were resuspended in 20 ml buffer A1. For solubilization of membrane proteins, *n*-dodecyl-*β*-D-maltopyranoside (DDM) was added to the membrane at a concentration of 1 mg/µg protein. To stabilize the Rnf complex, 5 µM flavin mononucleotide (FMN) was added and the membrane solution was stirred at 4° C for 2 h. Solubilized proteins were then separated from the membrane fraction by ultracentrifugation (130,000 ×*g*, 45 min, 4 °C). After that, proteins were diluted in buffer A2 (50 mM Tris–HCl, pH 8.0, 20 mM MgSO_4_, 5 µM FMN, 0.02% DDM, 2 mM DTE, and 4 µM resazurin) to decrease the initial DDM concentration to 0.5%. An incubation of the mixture together with 4 ml of nickel–nitrilotriacetic acid (Ni^2+^-NTA) material (Qiagen, Hilden, Germany) for 45 min at 4 °C followed. For further purification, bound proteins were washed with 40 ml wash buffer (50 mM Tris–HCl, 20 mM imidazole, pH 8.0, 20 mM MgSO_4_, 5 µM FMN, 0.02% DDM, 2 mM DTE, and 4 µM resazurin) and the Rnf complex was eluted in 4 ml steps in elution buffer (50 mM Tris–HCl, 150 mM imidazole, pH 8.0, 20 mM MgSO_4_, 5 µM FMN, 0.02% DDM, 2 mM DTE, and 4 µM resazurin).

### Measurements of ferredoxin:NAD oxidoreductase activity

Except stated otherwise, ferredoxin:NAD oxidoreductase activity was measured as described before [38]. Briefly, FNO activity was measured in 1.8 ml anoxic cuvettes (Glasgerätebau Ochs, Bovenden, Germany), and sealed with rubber stoppers under an CO atmosphere at 30 °C in buffer 20 mM Tris–HCl, pH 7.7, 2 mM DTE, and 4 µM resazurin. Before addition of the enzyme, ferredoxin from *C. pasteurianum* [39] was prereduced with purified CODH/ACS from *A. woodii* [21]. The reaction was started by addition of NAD^+^. The formation of NADH was observed and 1 unit (U) is defined as the reduction of 1 µmol NAD^+^/min.

### Preparation of proteoliposomes

Liposomes were prepared from phosphatidyl choline from soybeans by sonification. Proteoliposomes had to be prepared under anoxic conditions; therefore, 100 mg phosphatidyl choline were diluted in 10 ml buffer A1 (50 mM Tris–HCl, pH 8, 20 mM MgSO_4_) which was purged with N_2_ and contained 2 mM DTE and 4 µM resazurin for measurements with the Rnf complex. This phosphatidyl choline solution was sonified using a Sonifier (Bandelin Sonopuls, Bandelin, Deutschland) using the followed parameters: sonification time: 0.5 s, resting time 0.5 s, amplitude 30%, and total time 20 min. To destabilize the generated liposomes, 0.1% [w/v] DDM was added to the liposomes. After destabilization of the liposomes, protein was added in a protein-to-lipid ratio of 1:30 (w/w). For adhesion of liposomes and protein, the sample incubated under stirring for 30 min at RT. To stabilize the newly generated proteoliposomes, the detergent DDM was removed using biobeads (Bio-Beads SM-2, 20–50 mesh, Bio Rad, Germany). The detergent removal was performed successively by adding 80 mg/ml biobeads for 3 h at RT and adding 80 mg/ml for 1 h at RT, and a final removal step by adding 160 mg/ml biobeads over night at 4 °C while stirring. The biobeads were removed by filtration and the newly generated proteoliposomes were washed twice with buffer A using ultracentrifugation (Beckman Optima L90-K, 65.13TFT rotor, 130,000 x*g*, 30 min, 4 °C) before they were resuspended in 1 ml buffer A1.

### Measurement of ^22^Na^+^ translocation

Measurement of ^22^Na^+^ translocation by the Rnf complex was performed as described previously [30]. In brief, proteoliposomes were incubated with in buffer (100 mM Tris–HCl, pH 7.4, 100 mM maleic acid, 5 mM NaCl, and 5 mM MgCl_2_). Ferredoxin was added and reduced by the CODH under a CO atmosphere, NAD was added to a final concentration of 0.3 mM. Samples were taken and ^22^Na^+^ “trapped” inside the liposomes was separated from “external” ^22^Na^+^ using an electron exchange column. ^22^Na^+^ was detected by scintillation counting.

### Analytical methods

Protein concentrations were determined according to Bradford 1976 [40] or, in case of proteoliposomes, by the method of Lowry *et al.*, 1951 [41]. Proteins were separated in 12% SDS-polyacrylamide gels [42] and stained with Coomassie Brilliant Blue [43] or with silver [44]. Western blotting was done as described previously [45] using antibodies generated against the Rnf subunits RnfB, RnfC, and RnfG [46], the Na^+^-F_1_F_O_ ATP synthase AtpD, AtpB, AtpE, and AtpD [47], or the commercially available Penta-His antibody (Qiagen, Hilden, Germany). For determination of the molecular mass of the Rnf complex, a Native-PAGE was conducted under anoxic conditions. Size exclusion chromatography was also performed under anoxic conditions using a calibrated Superose 6 column (GE Life Sciences-Cytiva, Freiburg, Germany). Proteins were eluted from the column  using buffer A2-containing 50 mM Tris–HCl, pH 8.0, 20 mM MgSO_4_, 5 µM FMN, 0.02% DDM, 2 mM DTE, and 4 µM resazurin. Peptide mass fingerprinting by MALDI-TOF analysis was performed by the ‘Functional Genomics Center Zürich’ at the ETH Zurich, Switzerland, and results were analyzed using the Scaffold-Proteome Software version 4.10.0 (Proteome Software Inc., Portland, OR, USA).

## Supplementary Information

**Additional file 1: Sequence S1.** Sequence of plasmid pMTL_8312_Ptet_rnf containing the *rnf* operon from *A. woodii*. Underlined are the anhydrotetracycline inducible promoter region together with the gene coding for the *tet*-Repressor TetR. Letters in italics highlight the *rnf* gene cluster together with the N-terminal Strep-tag and the two His-tags on the C-terminus of RnfG and RnfB.

**Additional file 2: Figure S1.** Results of peptide mass fingerprinting (MALDI-TOF) analysis of the purified complex. The last column shows the total unique peptide count of the identified proteins.

## Data Availability

All data generated or analyzed during this study are included in this published article.

## Abbreviations

ATP: Adenosine triphosphate
Rnf: Ferredoxin: NAD oxidoreductase (*Rhodobacter* nitrogen fixation)
PMSF: Phenylmethylsulfonyl fluoride
DDM: *n*-dodecyl-*β*-D-maltopyranoside
FMN: Flavin mononucleotide
DTE: Dithioerythritol
NTA: Nitrilotriacetic acid
RT: Room temperature
SDS: Sodium dodecyl sulfate
PAGE: Polyacrylamide gel electrophoresis
MALDI-TOF: Matrix-assisted laser desorption/ionization-time of flight
PMF: Peptide mass fingerprinting
aTet: Anhydrotetracycline
TCS: 3,3',4',5'-tetrachlorosalicylanilide
ETH2120: N,N,N′,N′- tetracyclohexyl-1,2-phenylenedioxydiacetamide (sodium ionophore III)
