# Supplementary figures and images for: Homologous production, one-step purification, and proof of Na+ transport by the Rnf complex from Acetobacterium woodii, a model for acetogenic conversion of C1 substrates to biofuels
Source: Biotechnol Biofuels. 2020 Dec 21;13:208. doi: 10.1186/s13068-020-01851-4 (PMC7751120; doi:10.1186/s13068-020-01851-4)

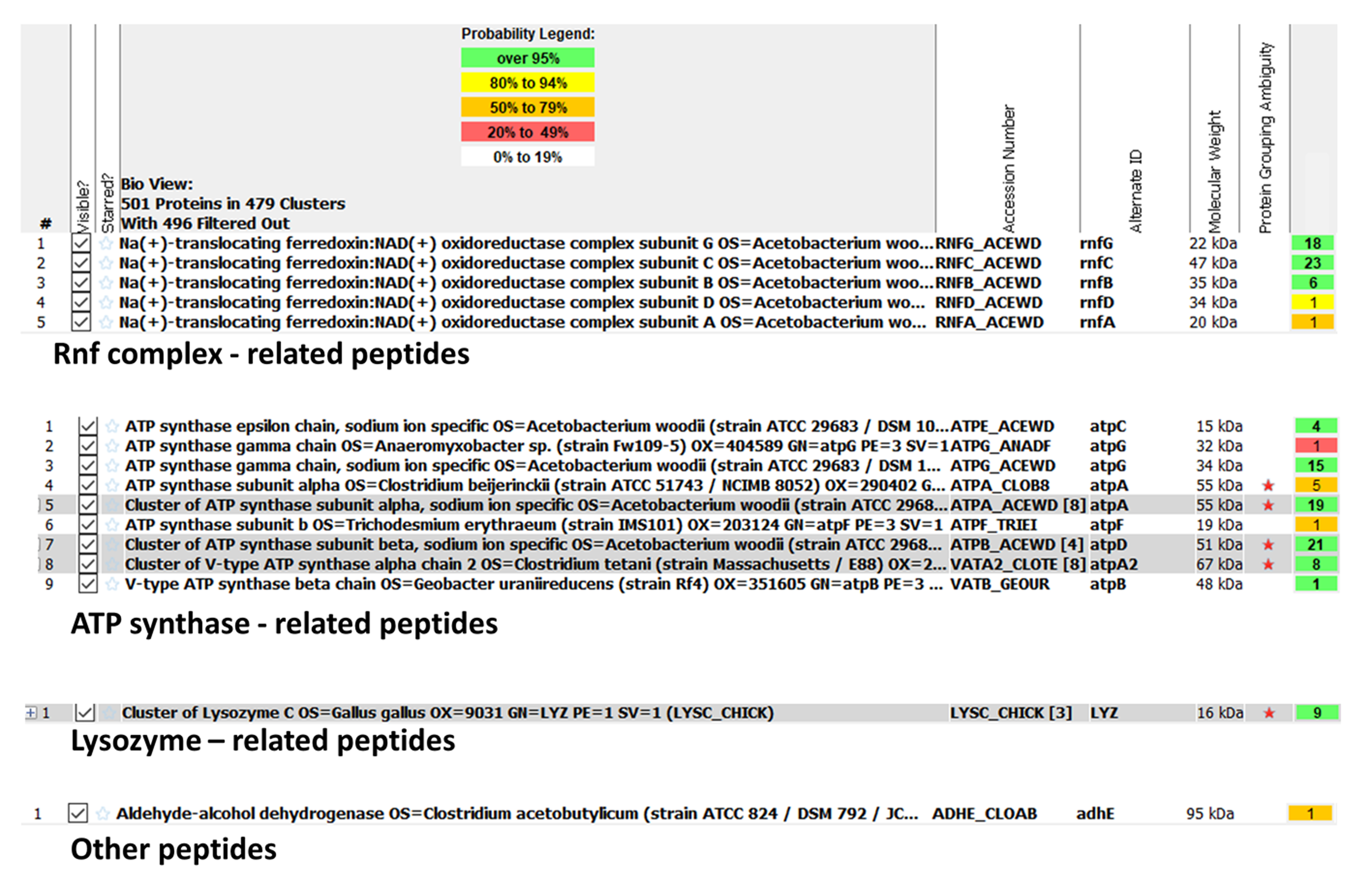

Supplement: Supplementary file 1 — Additional file 1: Sequence S1. Sequence of plasmid pMTL_8312_Ptet_rnf containing the rnf operon from A. woodii. Underlined are the anhydrotetracycline inducible promoter region together with the gene coding for the tet-Repressor TetR. Letters in italics highlight the rnf gene cluster together with the N-terminal Strep-tag and the two His-tags on the C-terminus of RnfG and RnfB. [file 13068_2020_1851_MOESM1_ESM.tif]
