# Supplementary material for: Homologous production, one-step purification, and proof of Na+ transport by the Rnf complex from Acetobacterium woodii, a model for acetogenic conversion of C1 substrates to biofuels
Source: Biotechnol Biofuels. 2020 Dec 21;13:208. doi: 10.1186/s13068-020-01851-4 (PMC7751120; doi:10.1186/s13068-020-01851-4)
Supplement: Supplementary file 2 — Additional file 2: Figure S1. Results of peptide mass fingerprinting (MALDI-TOF) analysis of the purified complex. The last column shows the total unique peptide count of the identified proteins. [file 13068_2020_1851_MOESM2_ESM.docx]

**Supplementary** **Sequence 1.** Sequence of plasmid pMTL_8312_Ptet_rnf containing the *rnf* operon from *A. woodii*. Underlined are the anhydrotetracycline inducible promoter region together with the gene coding for the *tet*-Repressor TetR. Letters in italics highlight the *rnf* gene cluster together with the N-terminal Strep-tag (highlighted in grey) and the two His-tags on the C-terminus of RnfG and RnfB ( highlighted in grey and in capital letters).

**pMTL_8312_Ptet_rnf**

cctgcaggataaaaaaattgtagataaattttataaaatagttttatctacaatttttttatcaggaaacagctatgaccgcggccgcttaagacccactttcacatttaagttgtttttctaatccgcatatgatcaattcaaggccgaataagaaggctggctctgcaccttggtgatcaaataattcgatagcttgtcgtaataatggcggcatactatcagtagtaggtgtttccctttcttctttagcgacttgatgctcttgatcttccaatacgcaacctaaagtaaaatgccccacagcgctgagtgcatataatgcattctctagtgaaaaaccttgttggcataaaaaggctaattgattttcgagagtttcatactgtttttctgtaggccgtgtacctaaatgtacttttgctccatcgcgatgacttagtaaagcacatctaaaacttttagcgttattacgtaaaaaatcttgccagctttccccttctaaagggcaaaagtgagtatggtgcctatctaacatctcaatggctaaggcgtcgagcaaagcccgcttattttttacatgccaatacaatgtaggctgctctacacctagcttctgggcgagtttacgggttgttaaaccttcgattccgacctcattaagcagctctaatgcgctgttaatcactttacttttatctaatctagacatcattaattcctcctttttgttgacattatatcattgatagagttatttgtcaaactagttttttatttcgatgccctggacttcatgaaaaactaaaaaaaatattgacactctatcattgatagagtataattaaaataagcttgatcgtagcgttaacagatctgagctcctgcagtaagggtcccgaagactcaatcgttagtcgacaggaggttaactcat*atgtggagccacccgcagttcgagaagaatgtaaaacatggaacctttaaggggggaatccacccaccttatagaaaggaaagtactgctgaagttcctttgggctttggcaaaaagccggaaatggtgatcataccaatgtcgcttcacattggcgcaccgtgtactccgattgtaaaaaaaggtgatacggtttttcttggacagcgggtcggagaaccaaatggatttgtttcagtacctgttcacgcaagtgtatcagggaaagtaattgcggtcgaagaacgaccacatgcatcgggtgatcgagtcatgtcagtcgttattgaatccgatggtttagatacgattgatccgtcgattaaaccgtatggaacattggaagacatggatgccgatgcgattaaaaaaatggttttaaacgccggaattgtcggtctcggaggggcaacctttcctacgcatgtaaagcttgcaataccaccagacaaaaaggtcgattgcgtcgtcttaaatggtgccgaatgtgaaccttatctaacggcagatcatcacttgatgacatcgcaagctgaaaaggttgtcatgggtttgaaattggcgatgaaatcagttggtgttgaaaaaggatttattggtgttgaagataacaaaaccgatgccatcgaagcattggtaaaagcgattggaaacgatagcagattggaagtttattcacttcatacaaaatacccccaaggggctgaaaaacagttgatcgctgccatcaccgggcgcgaagttccctctggagcattgccggcagatgctggtgtggtagtaatgaacgttggaaccgccgctcaaatagcagagtcaatgattacaggcttgccattatataaacgatatttaacctgtaccggagatgcgattaagaatcctcagaccatagagatccggattggcgtcccatttcagtcagtcattgatcaatgtggtggtttttcttctgaaccgggtaaagttatttccggtggaccgatgatgggcgttacacaatttgtaactgacattccggttatgaagggaacgtcaggaattctgtgtttaacgaaagaatcagcgaaaatagcgacgccatcaaattgcattcattgcggtaaatgtgttggggtatgtccaattcatttacagccacttaacattgccgagtattctcaacgaaatatgtgggataaatgtgagtcaaacaatgccatggattgcatcgaatgtggaagttgttcatatatctgtcctgcaaaacgaactttggtatcatccattcgggttgcaaagcgtgaaattatcgcgcaaagaagaaaaggaaattaggaggaatattgatgaatgaattaaatcttaccgtatcatcatcccctcatatccgggcaaataattccacagccagtattatgcaaaatgttattattgctttattacctgcattggctgtagcaggatatgtttttggattatgggcattggcacttgtggcaatttgtgtgatctcatcggtggctactgaagcggttattcaaaaactgcttaaaaaaccaattacggttaatgattggagtgcggttgtaacaggtgtgttgttagctttcaatttaccgattaatgcaccctggtggattggtgctgttggatcactttttgcaattgcaattgttaaacaatgttttggcggtttaggtcagaattttatcaataccggcacttgctgctcgagcctttttattggggcttcctggccaggtcatatgaccagcaccgcttatattccgttaactgatacagtcactacggcgacacctttagctttattaaaagctggagaaacaggtagtatgccttcaacgctggatttattcacgggattaaatggggtttacggttgtattggtgaaatatcggctttagctttgttaattggtggactttatcttatttataaaggcattattagttggcggattccaaccatttatttattgacaattgcgatctttgcgcttcttgtcggacaagatccaattgtgcatatggtatccggtggagtgatgttaggggcattttttatggcaactgactatgcttcgtcaccagttaccgccaaaggtcaaattatctatgcaatcggttgtggtttgattaccatgattattcgtctatatggtggatatccagaaggatgttcttattcaatactacttatgaatgttgcaacacctctaattgagcgcttcaccaaagaacgaatctatggtgtaaccaagatcaagaaggaggctaaagcataatggaaaccaaagaaaaagtacaaatcgattggaaagttgtttttaaacttggtttaattttatttgttatttctgcggtagcagcctgtgcgctggcattaaccaattacgtaacggcaggaaccattgaggaaatgaatgttcaaaccaatacggtggctcgtcaggaagtacttccgaaagcggctgattttgaagccgtacctgctaaagatgttgaaaaaatagcaagtgaaatcggaatggaaaaaccagaagaattgctggaagtatatatcggaaaaagtaatggtgaagttgttggatacaccgtaaaaaccggacctaccagtggttatgccggagaagttcaagtgctgaccggtatttccgcagatggcgttatcacgggcattacaattattaaaagtaatgaaaccccaggtttgggcgcgaaagcttcaggagtctggaatgatcaatttactgggaaatctgcaaaagaagaattggttgttgtaaaaggtaccacaaaagaaggaagtaacgagattcaggcaattactggttccaccattacttcaaaagcagttacttccggggtaaacatgtcgattcaagtttaccagaatttgtcgaaa*GGCGCCCACCATCACCATCACCA*ttaggagggaaaagtaaagatgaattttatgaagaatcttactcgaggaattattcgagaaaatcccacctttgtacttgtattggggatgtgtccgactttggcggttaccacatcagctatcaatggtatggggatggggctggcaacgatgctggtattgattggctcaaatgtcgctatctcggcattacggaaagtaataccggataatatccgaataccggcctttgttgttgttattgcatcgttcgttaccattgttgggatgttgatgaaagcttatgtaccagccttggatgcggcactgggaatttttattccattgattgttgttaactgtattattctggctcgagccgaagcgtttgctttttcgaatgggattgctgactcgtttgccgatgccgtcggaatgggtcttggttttacgttggcattaaccattttgggatcgatccgtgaaattttaggggctggcagtatttttggattttctttattcggggcagcttatgaaccggtattgttaatgatcctgcctcctggggcatttttaacccttggtcttttaattggtctcattaactggaaaacaaaaaaggcatagggggagatatcgatgactttaatttttataatgatcagtgcgatttttgttaataactttgtactgtcgcgatttttaggtatttgtccgtttttaggggtttctaaacaagtcgaaaccgctgttgggatgggggttgctgtaacctttgttatggctttagcttcggcgatcacttatgtggttcaatatgcaattctcgatccactgagtcttggttatcttcaaacaattgcatttattttaattattgcggctttggtgcaattagtagaaatgattatcaagaagtctagtccctctttgtatcaggcgctgggtgtatatcttccttttcttcctttgattacgaccaactgtgcggtacttggggtggcgttaattaatattcaaaatgaatacaacttcatcgaaaccatttttaatggtgttggtgcagcattaggtttcacgcttgcaattgttctttttgccggtattcgtgaacgacttgaaacttcggcagtgccaaaagcacttgaagggttcccgattgcattattaaccgctggtttaatggcgattgcatttttgggattctccggaatgaaattaggttaggaggatattgaaatgttaaatgcgattttagttccggtcggtatcctcggcgtattcggattaatttttggaattggtcttgctattgctgctaaggtatttgaagtatatgaagatccacgggtgcctttagttagagctgccctgcctggagcaaattgtggtggttgcggtttaccaggttgtgatgctttggcagccaatattgttggtggatctgcggcaattgatgcctgtcctgttggtggggcatcttgtgctgctgctgttgccgaaattatgggaatggaagcgggcagtgccgttaaaaaagtggccacggtcatttgtcagggaacctgcgagacggcacctaaccgcgccgaatattatggtgaaatggattgtcgtgaagccatgatcgcttcaggtggttcaaaaggctgtcgctatggttgtctgggatatggaacctgtaaagccgtttgcccatttgatgcaattgttattggcgaagatggtctgccaaaggtagatccggaaaaatgtacttcttgtggtaaatgcgtcgaggcttgtccaaaatcaattatgacgttagtccctgaagctcaggaagtaattgttaaatgtcataattttgataaaggtaaaattgcccgattatcatgtactacggcatgtattgcttgtggcgcttgcgttaaagcttgtcggtttgatgcaattacagtcgaaaataattgtgcgaaaattgattatgacaagtgtcgtcaatgttacgaatgtgttgataagtgtccgatgaattgtatttccggcgacgtggaatatggaaaatcaacggcttatattattgaagaaaattgtattgcgtgcggattgtgtgctaaaaattgtccggtaaacgcaattactggtgaaattaaaaaaccaccttatgttattgatcatgatatgtgcattggttgtggcatctgttttgacaaatgccgaaaaagtgcgattgaaatgcgtccaaataaaaccaaaCACCATCACCATCACCATtag*tgcgcagcctgaatggcgaatggcgctagcataaaaataagaagcctgcatttgcaggcttcttatttttatggcgcgccgccattatttttttgaacaattgacaattcatttcttattttttattaagtgatagtcaaaaggcataacagtgctgaatagaaagaaatttacagaaaagaaaattatagaatttagtatgattaattatactcatttatgaatgtttaattgaatacaaaaaaaaatacttgttatgtattcaattacgggttaaaatatagacaagttgaaaaatttaataaaaaaataagtcctcagctcttatatattaagctaccaacttagtatataagccaaaacttaaatgtgctaccaacacatcaagccgttagagaactctatctatagcaatatttcaaatgtaccgacatacaagagaaacattaactatatatattcaatttatgagattatcttaacagatataaatgtaaattgcaataagtaagatttagaagtttatagcctttgtgtattggaagcagtacgcaaaggcttttttatttgataaaaattagaagtatatttattttttcataattaatttatgaaaatgaaagggggtgagcaaagtgacagaggaaagcagtatcttatcaaataacaaggtattagcaatatcattattgactttagcagtaaacattatgacttttatagtgcttgtagctaagtagtacgaaagggggagctttaaaaagctccttggaatacatagaattcataaattaatttatgaaaagaagggcgtatatgaaaacttgtaaaaattgcaaagagtttattaaagatactgaaatatgcaaaatacattcgttgatgattcatgataaaacagtagcaacctattgcagtaaatacaatgagtcaagatgtttacataaagggaaagtccaatgtattaattgttcaaagatgaaccgatatggatggtgtgccataaaaatgagatgttttacagaggaagaacagaaaaaagaacgtacatgcattaaatattatgcaaggagctttaaaaaagctcatgtaaagaagagtaaaaagaaaaaataatttatttattaatttaatattgagagtgccgacacagtatgcactaaaaaatatatctgtggtgtagtgagccgatacaaaaggatagtcactcgcattttcataatacatcttatgttatgattatgtgtcggtgggacttcacgacgaaaacccacaataaaaaaagagttcggggtagggttaagcatagttgaggcaactaaacaatcaagctaggatatgcagtagcagaccgtaaggtcgttgtttaggtgtgttgtaatacatacgctattaagatgtaaaaatacggataccaatgaagggaaaagtataatttttggatgtagtttgtttgttcatctatgggcaaactacgtccaaagccgtttccaaatctgctaaaaagtatatcctttctaaaatcaaagtcaagtatgaaatcataaataaagtttaattttgaagttattatgatattatgtttttctattaaaataaattaagtatatagaatagtttaataatagtatatacttaatgtgataagtgtctgacagtgtcacagaaaggatgattgttatggattataagcggccggccagtgggcaagttgaaaaattcacaaaaatgtggtataatatctttgttcattagagcgataaacttgaatttgagagggaacttagatggtatttgaaaaaattgataaaaatagttggaacagaaaagagtattttgaccactactttgcaagtgtaccttgtacctacagcatgaccgttaaagtggatatcacacaaataaaggaaaagggaatgaaactatatcctgcaatgctttattatattgcaatgattgtaaaccgccattcagagtttaggacggcaatcaatcaagatggtgaattggggatatatgatgagatgataccaagctatacaatatttcacaatgatactgaaacattttccagcctttggactgagtgtaagtctgactttaaatcatttttagcagattatgaaagtgatacgcaacggtatggaaacaatcatagaatggaaggaaagccaaatgctccggaaaacatttttaatgtatctatgataccgtggtcaaccttcgatggctttaatctgaatttgcagaaaggatatgattatttgattcctatttttactatggggaaatattataaagaagataacaaaattatacttcctttggcaattcaagttcatcacgcagtatgtgacggatttcacatttgccgttttgtaaacgaattgcaggaattgataaatagttaacttcaggtttgtctgtaactaaaaacaagtatttaagcaaaaacatcgtagaaatacggtgttttttgttaccctaagtttaaacaagatgatcttcttgagatcgttttggtctgcgcgtaatctcttgctctgaaaacgaaaaaaccgccttgcagggcggtttttcgaaggttctctgagctaccaactctttgaaccgaggtaactggcttggaggagcgcagtcaccaaaacttgtcctttcagtttagccttaaccggcgcatgacttcaagactaactcctctaaatcaattaccagtggctgctgccagtggtgcttttgcatgtctttccgggttggactcaagacgatagttaccggataaggcgcagcggtcggactgaacggggggttcgtgcatacagtccagcttggagcgaactgcctacccggaactgagtgtcaggcgtggaatgagacaaacgcggccataacagcggaatgacaccggtaaaccgaaaggcaggaacaggagagcgcacgagggagccgccagggggaaacgcctggtatctttatagtcctgtcgggtttcgccaccactgatttgagcgtcagatttcgtgatgcttgtcaggggggcggagcctatggaaaaacggctttgccgcggccctctcacttccctgttaagtatcttcctggcatcttccaggaaatctccgccccgttcgtaagccatttccgctcgccgcagtcgaacgaccgagcgtagcgagtgggccccctgcttcggggtcattatagcgattttttcggtatatccatcctttttcgcacgatatacaggattttgccaaagggttcgtgtagactttccttggtgtatccaacggcgtcagccgggcaggataggtgaagtaggcccacccgcgagcgggtgttccttcttcactgtcccttattcgcacctggcggtgctcaacgggaatcctgctctgcgaggctggccggctaccgccggcgtaacagatgagggcaagcggatggctgatgaaaccaagccaaccaggaagggcagcccacctatcaaggtgtactgccttccagacgaacgaagagcgattgaggaaaaggcggcggcggccggcatgagcctgtcggcctacctgctggccgtcggccagggctacaaaatcacgggcgtcgtggactatgagcacgtccgcgagctggcccgcatcaatggcgacctgggccgcctgggcggcctgctgaaactctggctcaccgacgacccgcgcacggcgcggttcggtgatgccacgatcctcgccctgctggcgaagatcgaagagaagcaggacgagcttggcaaggtcatgatgggcgtggtccgcccgagggcagagccatgacttttttagccgctaaaacggccggggggtgcgcgtgattgccaagcacgtccccatgcgctccatcaagaagagcgacttcgcggagctggtgaagtacatcaccgacgagcaaggcaagaccgatcgggccc
